# Integrated bulk and single-cell transcriptomics unveil the role of AHCY in cell cycle regulation of endometrial cancer

**DOI:** 10.3389/fonc.2026.1770032

**Published:** 2026-08-20

**Authors:** Haiyang Zhang, Li Du, Wei Ren, Jingli Sun, Lipeng Pei, Yao Fu

**Affiliations:** 1 Department of Oncology and Hematology, Dongping County Peoples Hospital, Taian, China; 2 Department of Obstetrics and Gynecology, General Hospital of Northern Theater Command, Shenyang, China

**Keywords:** AHCY, endometrial cancer, macrophages, prognostic analysis, single-cell RNA sequencing analysis

## Abstract

**Background:**

Although AHCY has been implicated in cancer progression, its specific role in endometrial cancer (EC) remains poorly understood. This study aimed to elucidate the oncogenic role of AHCY in EC and its association with patient prognosis.

**Methods:**

The study was initiated by analyzing AHCY expression, prognostic relevance, and immunotherapy associations across multiple cancers in TCGA Pan-Cancer project. The connection between AHCY expression and patient survival was then assessed through Kaplan-Meier survival analysis within EC datasets. Further investigations included functional annotation of AHCY, its correlation with the tumor immune microenvironment, and screening of potential targeting drugs. Single-cell RNA sequencing data were interrogated to identify key cell types associated with AHCY expression. Finally, experimental validation was conducted using clinical specimens and cell lines to examine AHCY expression, and functional consequences of AHCY knockdown—including effects on cell function, cell cycle, and apoptosis—were systematically assessed.

**Results:**

AHCY was associated with cancer prognosis, cell cycle, and response to immunotherapy, and was correlated with clinical stage and prognosis in EC. Pathways such as cell cycle and DNA replication were activated in the samples with high expression of AHCY. Most immune cells exhibit reduced infiltration in the AHCY high-expression group. Macrophages were selected as the key cell type, which exhibited a significant difference in AHCY expression between EC and control samples. The expression of AHCY was remarkably upregulated in clinical EC tissues or Ishikawa cells. AHCY knockdown exerted anti-tumor effects by concurrently impairing proliferative and metastatic capacities, enhancing apoptotic cell death, and arresting cell cycle. Folic acid, computationally identified as an AHCY-associated candidate, exerted a marked anti-proliferative effect on Ishikawa cells, demonstrating dose-dependent growth inhibition.

**Conclusion:**

AHCY is associated with patient prognosis and malignant phenotypes in EC, and macrophages represent a potentially relevant AHCY-expressing cell population within the tumor microenvironment. This study sheds light on EC pathogenesis and suggests potential for targeted therapy.

## 1 Introduction

Endometrial cancer (EC) is the most common gynecological cancer in high-income countries, and its incidence is rising globally (1). In 2022, there were 420,242 newly diagnosed cases and 97,704 deaths worldwide (2). EC originates in the uterine lining and is histologically classified as endometrioid carcinoma (~80% of cases) or non-endometrioid carcinoma (~20%) (3). EC incidence and mortality have risen for decades, fueled by overweight/obesity and lagging therapeutic progress (4, 5). Despite advances in surgery, chemotherapy, targeted agents, and immunotherapy, treatment options for metastatic or recurrent EC remain limited (6). Therefore, elucidating the key biological mechanisms underlying EC progression and developing novel therapeutic strategies based on this understanding holds significant clinical and scientific importance.

S-adenosylhomocysteine hydrolase (AHCY/SAHH) catalyzes the reversible hydrolysis of S-adenosylhomocysteine (SAH) to adenosine and homocysteine (7, 8), sustaining cellular transmethylation by maintaining a favorable S-adenosylmethionine (SAM):SAH ratio (9). Its physiological importance is evidenced by the rare multisystem disorder known as human AHCY deficiency (10, 11). Increasing evidence implicates AHCY in cancer biology: context-dependent alterations in AHCY expression or activity reshape epigenetic states and DNA-damage responses and influence cell-cycle progression, proliferation, and motility (12–14). Moreover, beyond SAH clearance, AHCY-derived adenosine modulates intracellular metabolism and purinergic signaling that may support immunosuppressive tumor microenvironments (15). Collectively, AHCY, as a central node integrating metabolism, epigenetics, and immune regulation, demonstrates significant diagnostic and therapeutic potential.

Given that AHCY links transmethylation metabolism with epigenetic regulation, cell-cycle progression, and immune modulation—all of which are closely involved in EC development—we selected AHCY as the focal molecule of this study. However, its clinical and functional significance in EC remains unclear. We hypothesized that AHCY is aberrantly upregulated in EC, is associated with poor patient prognosis, and promotes malignant progression by sustaining cell-cycle activity and suppressing apoptosis. To test this hypothesis, we integrated pan-cancer, bulk transcriptomic, and single-cell analyses with validation in clinical specimens and EC cells. We further evaluated the effects of AHCY knockdown on cell viability, migration, invasion, apoptosis, and cell-cycle progression and explored its potential as a therapeutic target in EC.

## 2 Materials and methods

### 2.1 Data collection

The Xena database (https://xenabrowser.net/datapages/) of the University of California Santa Cruz (UCSC) provided the RNA-seq and phenotypic data for the GDC TCGA UCEC cohort (19 datasets, n = 585), of which 535 cancer cases had complete overall survival (OS) information. The National Center for Biotechnology Information (NCBI) database (https://www.ncbi.nlm.nih.gov/) contributed the single-cell RNA sequencing (scRNA-seq) data. The dataset SRP349751, derived from endometrial tissue (5 cancer cases and 5 controls; n=10), was integrated with data from 5 tumor samples (patient 1-5) from the GSE173682 dataset (16). The combined dataset, comprising 10 cancer cases and 5 controls, was then subjected to an integrated single-cell analysis. The TCGA Pan-Cancer project (https://portal.gdc.cancer.gov/projects/) delivered the data for various human cancer types.

### 2.2 Pan-cancer expression profiling and prognostic correlation of AHCY

Utilizing data from the TCGA Pan-Cancer project, AHCY expression across various human cancers and their matched adjacent normal tissues was analyzed, with the results visualized in box plots. Protein expression levels of AHCY were analyzed employing the UALCAN database (https://ualcan.path.uab.edu/). AHCY expression data across human cancer cell lines were sourced from the DepMap portal (Expression Public 25Q3), comprising log_2_(TPM + 1) normalized RNA-seq values for 1,699 cell lines in Cancer Cell Line Encyclopedia (CCLE). The prognostic significance of AHCY in pan-cancer was evaluated via univariable Cox regression analyses with respect to OS, disease-specific survival (DSS), disease-free interval (DFI), and progression-free interval (PFI), implemented applying the survival (v3.4-0) package (17).

### 2.3 Copy number alteration (CNA) and RNA modifications of AHCY in pan-cancer

By integrating CNA and gene expression data from the pan-cancer cohort, AHCY alteration levels were compared among samples with neutral, gain, and loss copy number status for each cancer type. The Kruskal-Wallis test was applied for multi-group comparisons, followed by Wilcoxon test for differences between two groups. The relationship between AHCY and four DNA methyltransferases (DNMT1, TRDMT1, DNMT3A, and DNMT3B) in pan-cancer was assessed through correlation analysis. To further investigate the relationship between AHCY and RNA modifications, Pearson correlation analysis was performed between AHCY and a set of 44 genes involved in m6A (n = 21), m5C (n = 13), and m1A (n = 10) regulation based on their expression data. Additionally, the global m6A modification level in EC tissues was evaluated using a dot-blot assay. To investigate the relationship between AHCY and genomic instability markers, we assessed its connection with microsatellite instability (MSI) and tumor mutational burden (TMB).

### 2.4 Associations of AHCY with cell cycle pathway activity and immune checkpoints in pan-cancer

Pearson correlation analysis was executed between AHCY expression and that of 60 immune checkpoints utilizing data from the TCGA Pan-Cancer project (18). To evaluate the association between cell cycle pathway activity and AHCY expression in pan-cancer, ssGSEA was applied to derive pathway scores, followed by cross-cancer correlation analysis utilizing the psych (v2.2.9) package.

### 2.5 Prognostic and clinical relevance of AHCY in endometrial cancer

Within the TCGA UCEC cohort, tumor patients were stratified into high- and low-AHCY expression cohorts with the optimal cut-off. Differences in OS, DSS, PFI, and DFI between the two cohorts were subsequently analyzed. Subsequently, receiver operating characteristic (ROC) curves were plotted to assess the predictive performance of AHCY expression for 1-, 2-, and 3-year OS in EC patients. Furthermore, the association between AHCY expression levels and clinical stage was evaluated.

### 2.6 Differential expression analysis and functional annotation analysis

Raw TCGA-UCEC count data were normalized using estimateSizeFactors, and differential expression between the high- and low-AHCY groups was analyzed using the negative binomial model in DESeq2 (v1.36.0). Genes with |log_2_FC| > 1 and FDR < 0.05 were considered DEGs (19). DEGs were applied for Kyoto Encyclopedia of Genes and Genomes (KEGG) enrichment analysis via clusterProfiler (v4.2.2) package (20), as well as Gene Ontology (GO) annotation, to delineate biological themes and pathways (*P* < 0.05).

### 2.7 Gene set enrichment analysis

GSEA was implemented via the clusterProfiler package. The gene list was ranked based on the log_2_FC values of DEGs between AHCY expression cohorts. Pathways were considered significantly enriched when meeting the criteria of |NES| > 1 and *P* < 0.05. Additionally, GSEA was implemented adopting the correlation coefficients between AHCY and all other genes in the TCGA UCEC cohort as the ranking metric (*P* < 0.05). The analysis utilized the c2.cp.kegg.v2023.2.Hs.symbols.gmt gene set, which was acquired from the MSigDB database (http://www.gsea-msigdb.org/gsea/msigdb).

### 2.8 The impact of AHCY on immune microenvironment and immunotherapy in EC

The abundance of immune cell infiltration in the disease cohort within the TCGA UCEC cohort was quantified by utilizing the ssGSEA algorithm. Subsequently, Wilcoxon tests were employed to compare the infiltration levels of each immune cell type between the high- and low-AHCY expression cohorts (*P* < 0.05). Subsequently, the Tumor Immune Dysfunction and Exclusion (TIDE) analysis was applied to the two AHCY expression cohorts. This framework evaluates the combined effects of cytotoxic T-lymphocyte infiltration and immunosuppressive factors to estimate the potential clinical efficacy of immune checkpoint blockade.

### 2.9 Immunohistochemical validation

The expression patterns of AHCY in both normal and EC tissues were retrieved in the Human Protein Atlas (HPA) database (https://www.proteinatlas.org/).

### 2.10 scRNA-seq analysis

The integrated single-cell dataset was processed by the Seurat (v4.1.0) package (21). Initial filtering removed genes detected in fewer than 3 cells. Subsequently, cells with 1) >20% mitochondrial reads, 2) nFeature_RNA ≤ 200 or ≥ 4,000, and 3) nCount_RNA ≤ 200 or ≥ 15,000 were eliminated. Data were normalized using LogNormalize (scale factor = 10,000), and the top 2,000 highly variable genes were identified using the vst method. Batch effects were corrected using Harmony (v0.1.0; theta = 2; maximum iterations = 20). The first 30 principal components (PCs) selected by the JackStraw test were used for Uniform Manifold Approximation and Projection (UMAP) and clustering at resolution 0.8, as determined using clustree. Cluster markers were identified using FindAllMarkers with the Wilcoxon test (|log_2_FC| > 0.5 and FDR < 0.05), and cell types were annotated using the CellMarker database. Furthermore, the DoubletFinder package (v2.0.4) was applied to detect and remove potential doublets (22). The expression and distribution of AHCY were examined across various cell types, comparing tumor samples with normal samples. Key cell types were selected based on AHCY expression levels.

### 2.11 Cell-cell communication and pseudotime trajectory analyses

Cell-cell communication was analyzed by examining ligand-receptor interactions across different cell types utilizing CellPhoneDB (23). The interaction signals for each cell group were visualized to depict the ligand-receptor pairs present in different samples. To reconstruct the cellular developmental trajectory, pseudotime analysis of key cells was applied using Monocle (v2.26.0) package (24). The expression profile of AHCY was subsequently examined across pseudotime.

### 2.12 CRISPR dependency analysis

AHCY dependency data for uterine-derived cell lines were obtained from the DepMap portal (Public 26Q1 release). Chronos gene-effect scores were available for 34 cell lines, including 28 endometrial carcinoma cell lines, with more negative scores indicating greater impairment of cellular fitness following gene knockout.

### 2.13 Drug sensitivity profiling and potential drug identification

To identify potential therapeutic agents, the half-maximal inhibitory concentration (IC50) values of 198 compounds from the GDSC portfolio were predicted adopting the oncoPredict (v0.2) package (25). Differential drug sensitivity between the two expression groups was assessed by comparing these IC50 values via the Wilcoxon test. Potential therapeutic agents targeting AHCY were identified in the Comparative Toxicogenomics Database (CTD; https://ctdbase.org/). A gene-drug interaction network was established, from which non-toxic agents were selected for subsequent molecular docking through AutoDock Vina (26). The 3D structures of the selected non-toxic agents and AHCY were retrieved in the PubChem database (https://pubchem.ncbi.nlm.nih.gov) and Protein Data Bank (PDB, http://www.rcsb.org/pdb/). respectively. Both structures were prepared for docking applying PyMOL software, which involved removing water molecules and native ligands from the protein structure. Further detection was carried out through cell experiments to examine the effects of the above-mentioned non-toxic agents on the viability of EC cells, as well as the effects of AHCY expression in EC cells.

### 2.14 Cell culture and sample collection

Human endometrial epithelial cells (hEEC) and the human EC cell lines (Ishikawa, HEC-1-B, and AN3CA) were procured from Procell Life Science&Technology Co.,Ltd. (Wuhan, China). Cells were cultured at 37 °C with 5% CO_2_ in their respective media with 1% penicillin/streptomycin: hEEC in DMEM with 10% FBS; Ishikawa in RPMI-1640 with 10% FBS; HEC-1-B in MEM with 10% FBS; and AN3CA in DMEM with 15% FBS. Specimens comprising five paired EC tumors and matched adjacent noncancerous endometrium were obtained from pathologically confirmed patients at the General Hospital of Northern Theater Command. The study was approved by the Institutional Ethics Committee (Approval No.: Y (2025) 260), and written informed consent was obtained from all participants.

### 2.15 Reverse transcription-quantitative polymerase chain reaction

Utilizing RT-qPCR, the mRNA expression level of AHCY was examined in various cell lines or tissues. The FastPure Complex Tissue/Cell Total RNA Isolation Kit (Vazyme Biotech) was applied to extract total RNA from cells or tissues. The purity of the isolated RNA was assessed with a NanoDrop 500 microspectrophotometer, and only samples with an OD260/OD280 ratio greater than 1.8 were applied for subsequent analysis. The ABScript III RT Master Mix for RT-qPCR with gDNA Remover (ABclonal) was adopted to carry out reverse transcription. Subsequent quantitative PCR amplification was carried out with the Genious 2X SYBR Green Fast RT-qPCR Mix (ABclonal) under standard cycling conditions, with GAPDH as an internal reference (**Supplementary Table 1**). Relative expression was computed via 2^–ΔΔCt, with technical triplicates and biological replicates for cell lines.

### 2.16 Establishment and validation of AHCY-knockdown Ishikawa cell models

To knock down AHCY expression, Ishikawa cells were transfected with three different short hairpin RNA (shRNA) constructs that targeted various AHCY regions (**Supplementary Table 1**). RT-qPCR and Western blot analysis were subsequently employed to confirm the effectiveness of knockdown at the mRNA and protein levels, respectively. The functional impact of AHCY knockdown on Ishikawa cells was subsequently assessed. Cell viability was assessed employing the CCK-8 assay. Cellular migration and invasion capabilities were determined by wound healing and Transwell invasion assays, respectively.

### 2.17 Western blot

Total protein from Ishikawa cells was extracted utilizing the Beyotime RIPA Lysis buffer and quantified applying the Beyotime BCA protein kit. The samples were separated via SDS-PAGE following protein denaturation, and then they were put onto a PVDF membrane. 5% skim milk made in TBST was employed to block the membrane. After washing, a primary antibody (AHCY, 1: 1000, abways, CY8770; GAPDH, 1: 10000, Proteintech, 10494-1-AP) was added to the membrane and incubated for the entire night at 4 °C. Following an additional washing step, the membrane was incubated with a secondary antibody (HRP-goat anti-mouse/rabbit IgG (H+L), Invitrogen, 31430/31460) diluted at a ratio of 1:5000 in TBST. Finally, the blots were treated with ECL reagent and immediately visualized adopting a chemiluminescence imaging system.

### 2.18 Cell viability assay

Cells were inoculated at 2,000–4,000 per well in a 96-well plate, and cell viability was assessed at 0, 24, 48, and 72 hours. At each time point, 10 µL of CCK-8 solution was added to the cultures, which were then incubated for an additional 1 hour at 37 °C. The absorbance at 450 nm (OD_450_) was measured using a microplate reader.


$$\text{Viability }(\%)=({\text{OD}}_{\text{treatment}}-{\text{OD}}_{\text{blank control}})/({\text{OD}}_{\text{negative control}}-{\text{OD}}_{\text{blank control}})\times 100\%$$

### 2.19 Cell migration capacity assay

The cells (6 x 10^4^ per well) were grown until they reached 90% confluence after being sown onto 6-well plates. A sterile pipette tip was inserted to make a scratch. Images of the impacted areas were obtained at 0 and 24 hours in order to monitor cell migration. The migration rate based on the wound area was calculated using the following formula:


Migration rate (%)=(Area at 0h−Area at t h)/Area at 0h×100%

### 2.20 Cell invasion assay

Transwell plates were utilized for cell invasion tests. To evaluate their capacity for invasion, 6 x 10^4^ cells per well were sown into the upper chamber and cultivated in an incubator. A moistened cotton swab was used to carefully remove any non-invading cells that remained on the membrane’s upper surface after a 24- to 48-hour incubation period. After 20–30 minutes of 4% paraformaldehyde immobilization, the invasive cells on the lower surface were colored with 0.1% crystal violet. After that, photos were taken under a microscope and subjected to statistical analysis.

### 2.21 Effect of AHCY on apoptosis and cell cycle progression in Ishikawa cells

Apoptosis and cell cycle distribution were analyzed in both control and AHCY-knockdown Ishikawa cells employing a Cell Cycle and Apoptosis Analysis Kit (Beyotime). Red fluorescence and light scattering properties were then measured employing flow cytometry on the stained cells at an excitation wavelength of 488 nm. Western blot was also adopted to assess the expression of cell cycle-related proteins in both control and AHCY-knockdown Ishikawa cells.

### 2.22 RNA m6A modification level in EC tissues

The m6A modification level in clinical tissue samples was detected by a dot-blot assay. Briefly, UV cross-linking (120 mJ/cm2, 254 nm) was employed to immobilize whole RNA on a membrane. An anti-m6A monoclonal antibody (1: 500, PROTEINTECH, 68055-1-Ig) and an HRP-goat anti-mouse IgG (H+L) secondary antibody (Invitrogen, 31430, 1: 5,000) served to incubate the membrane after it had been blocked in 5% non-fat milk. The signal was visualized with ECL substrate and documented utilizing a chemiluminescence imaging system.

### 2.23 Statistical analysis

All bioinformatic analyses were performed using R. Intergroup differences were evaluated using the Wilcoxon test. For multiple comparisons, *P* values were adjusted using the Benjamini-Hochberg method, with FDR < 0.05 considered significant. Effect sizes were reported as log_2_FC, Pearson’s r, hazard ratios, or normalized enrichment scores, as appropriate. Image data were quantified using Image J, and experimental data were analyzed by one-way ANOVA using GraphPad Prism, with *P* < 0.05 considered significant.

## 3 Results

### 3.1 AHCY was associated with cancer prognosis

**Figure 1:**
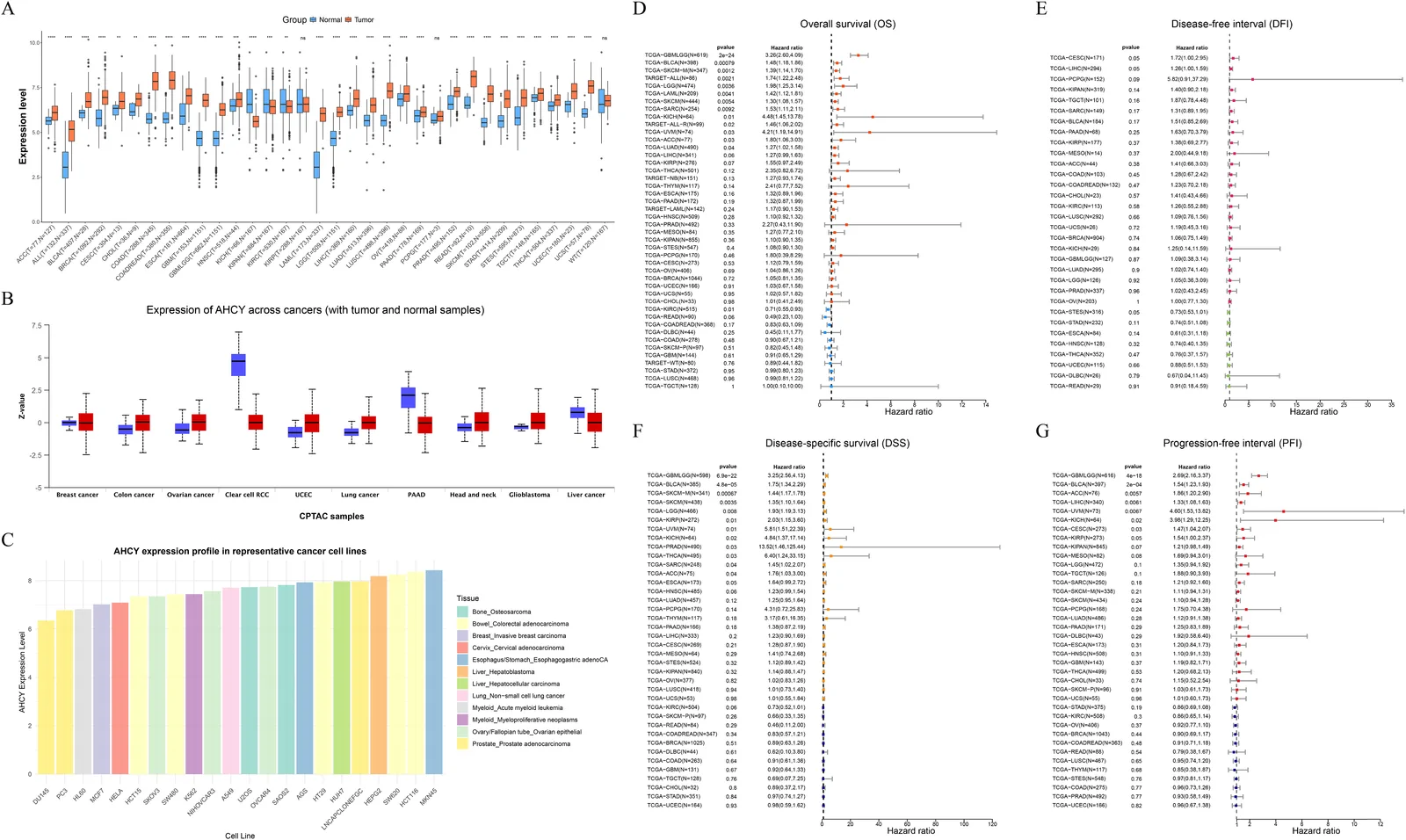
Association of AHCY with cancer prognosis. **(A)** Box plot shows AHCY expression in 34 types of cancer. **(B)** Box plot showing AHCY protein expression in 10 types of cancer. **(C)** Bar plot showing AHCY expression in representative cancer cell lines. **(D–G)** Univariable Cox analysis of AHCY expression with overall survival (OS) **(D)**, and disease-free interval (DFI) **(E)**, disease-specific survival (DSS) **(F),** progression-free interval (PFI) **(G)** in 31 types of cancer. ns, not significant; **P < 0.01, ***P < 0.001, ****P < 0.0001.

Analysis of the TCGA Pan-Cancer project revealed that AHCY expression was markedly altered in 31 types of cancer, such as in EC, breast cancer, and colon adenocarcinoma (COAD) (**Figure 1A**). Differential protein expression of AHCY was observed in several cancers, including renal cell carcinoma (RCC), EC, and pancreatic adenocarcinoma (PAAD) (**Figure 1B**). Further analysis revealed that AHCY was expressed in a variety of cancer cell lines originating from breast, lung, prostate, and pancreatic tissues (**Figure 1C**). Univariable Cox analysis indicated significant associations between AHCY expression and patient prognosis across multiple cancer types (**Figures 1D–G**). It was associated with OS in 13 cancers, with DSS in 12 cancers, and with PFI in 7 cancers (e.g., bladder urothelial carcinoma and glioma).

### 3.2 AHCY was associated with genomic instability, cell cycle progression, and immune regulation

**Figure 2:**
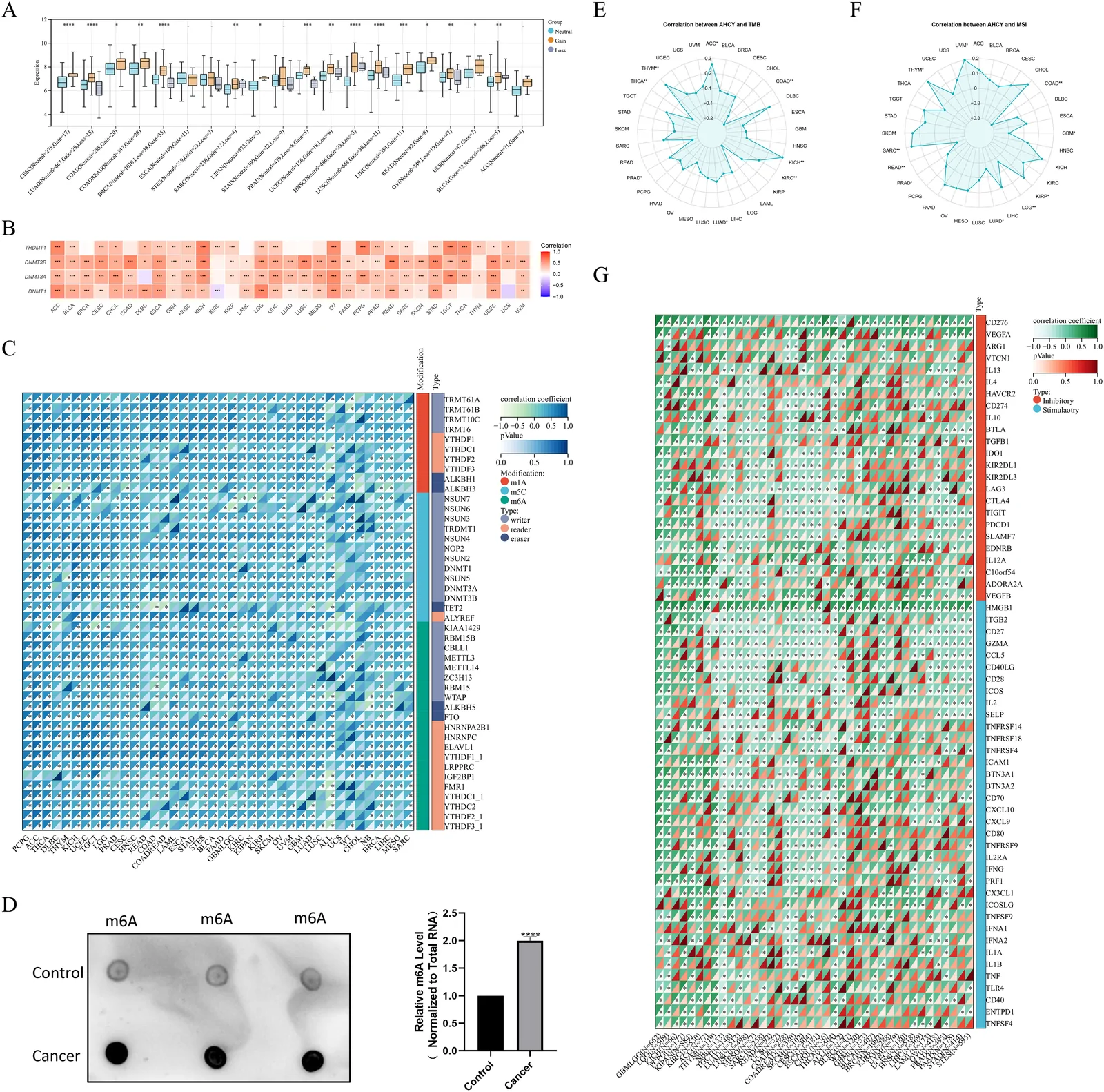
Association of AHCY with response to immunotherapy and genomic changes. **(A)** AHCY expression in 20 cancer types when tumors were stratified by CNA status (neutral, gain, loss). **(B)** Correlation heatmap of four DNA methyltransferases and AHCY expression in 33 cancer types. **(C)** Correlation heatmap of RNA modification regulators and AHCY expression in 40 cancer types. **(D)** The m6A modification level in clinical EC tissues compared with paired adjacent normal tissues. **(E)** Correlation between AHCY expression and tumor mutational burden (TMB). **(F)** Correlation between AHCY expression and microsatellite instability (MSI). **(G)** Correlation heatmap of immune checkpoints and AHCY expression in 38 cancer types. -, not significant; *P < 0.05, **P < 0.01, ***P < 0.001, ****P < 0.0001.

**Figure 3:**
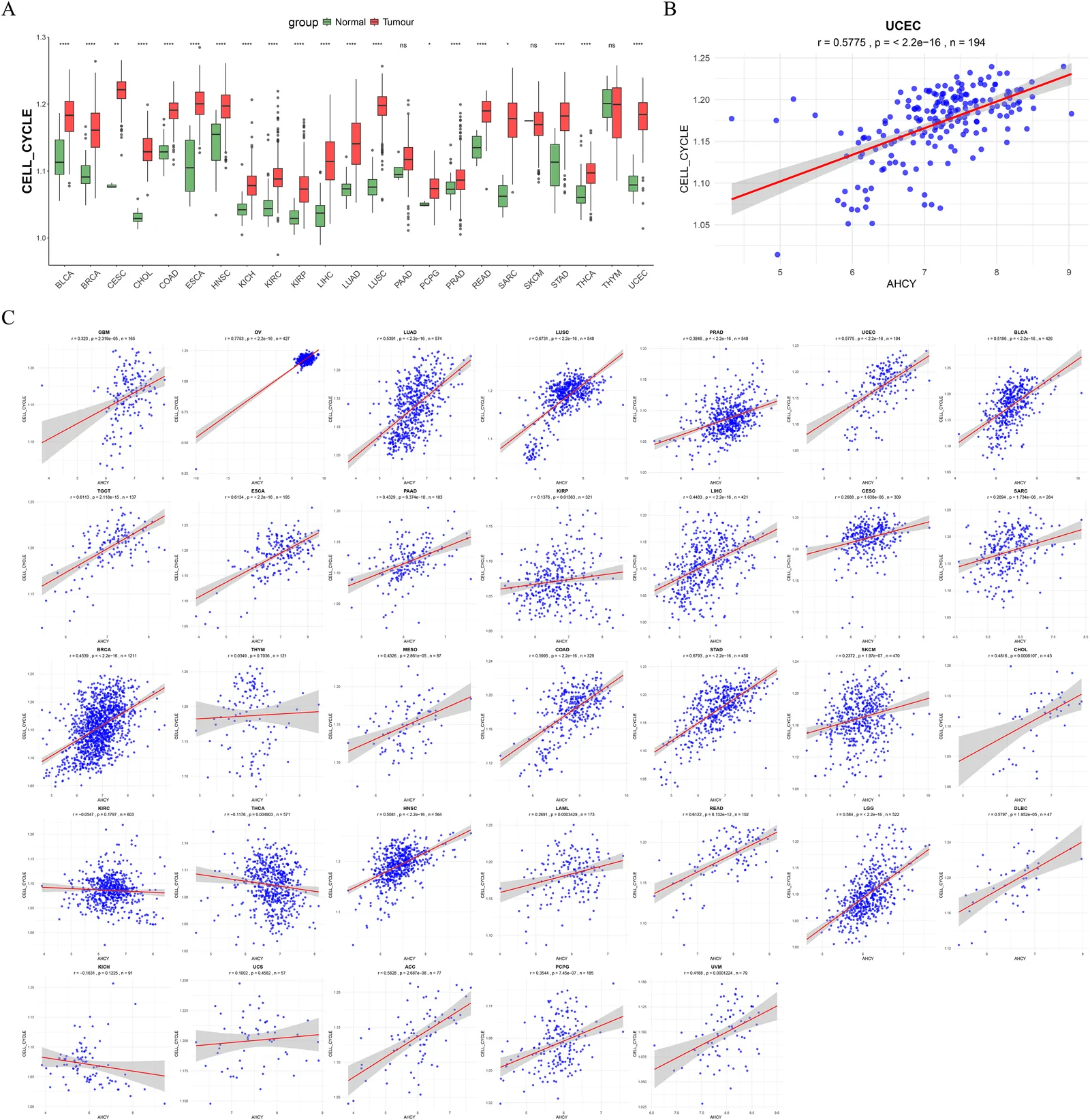
The association between cell cycle pathway activity and AHCY expression in pan-cancer. **(A)** Box plot showing cell cycle activity scores in pan-cancer. **(B)** Correlation of cell cycle pathway activity and AHCY expression in endometrial cancer (EC). **(C)** Correlation of cell cycle pathway activity and AHCY expression in other cancer types. ns, not significant; *P < 0.05; **P < 0.01; ***P < 0.001; ****P < 0.0001.

AHCY expression significantly differed in 16 (e.g., EC and COAD) of 20 cancer types when tumors were stratified by CNA status (neutral, gain, loss), as determined through integrated genomic profiling (**Figure 2A**). AHCY demonstrated a consistent positive correlation with four DNA methyltransferases (DNMT1, TRDMT1, DNMT3A, and DNMT3B) across various cancers, most notably showing a strong significant correlation in EC (**Figure 2B**). AHCY exhibited prevalent co-expression with RNA modification regulators, showing positive correlations in almost 31 cancer types, as exemplified by TRMT61A, YTHDF3_1, LRPPRC and YTHDF1 (**Figure 2C**). Comprehensive m6A profiling revealed a remarkable upregulation of the m6A modification in endometrial carcinoma tissues compared to paired non-cancerous tissues (**Figure 2D**). Notably, AHCY expression was correlated with TMB in several cancer types including but not limited to adrenocortical carcinoma, lung adenocarcinoma, and prostate adenocarcinoma, while its association with MSI was observed in cancers such as uveal melanoma and COAD (**Figures 2E, F**). Analysis of immune checkpoints revealed that AHCY demonstrated significant positive correlations with a substantial number of them across diverse cancer types, notably with HMGB1 and CD276 (**Figure 2G**). Further analysis revealed significant differences in cell cycle activity scores between multiple cancer types and corresponding normal controls. This disparity was particularly pronounced in EC, where the score differential was most marked (**Figure 3A**). A moderate positive correlation (r = 0.58, *P* < 0.05) was observed between AHCY expression and cell cycle activity in EC, with consistent associations also identified in other cancer types (**Figures 3B, C**). Based on these findings, AHCY emerges as a multi-faceted oncoprotein with a distinct pan-cancer profile encompassing dysregulated expression, epigenetic coordination, and significant associations with genomic instability, cell cycle progression, and immune regulation.

### 3.3 AHCY expression was correlated with clinical stage and prognosis in EC

**Figure 4:**
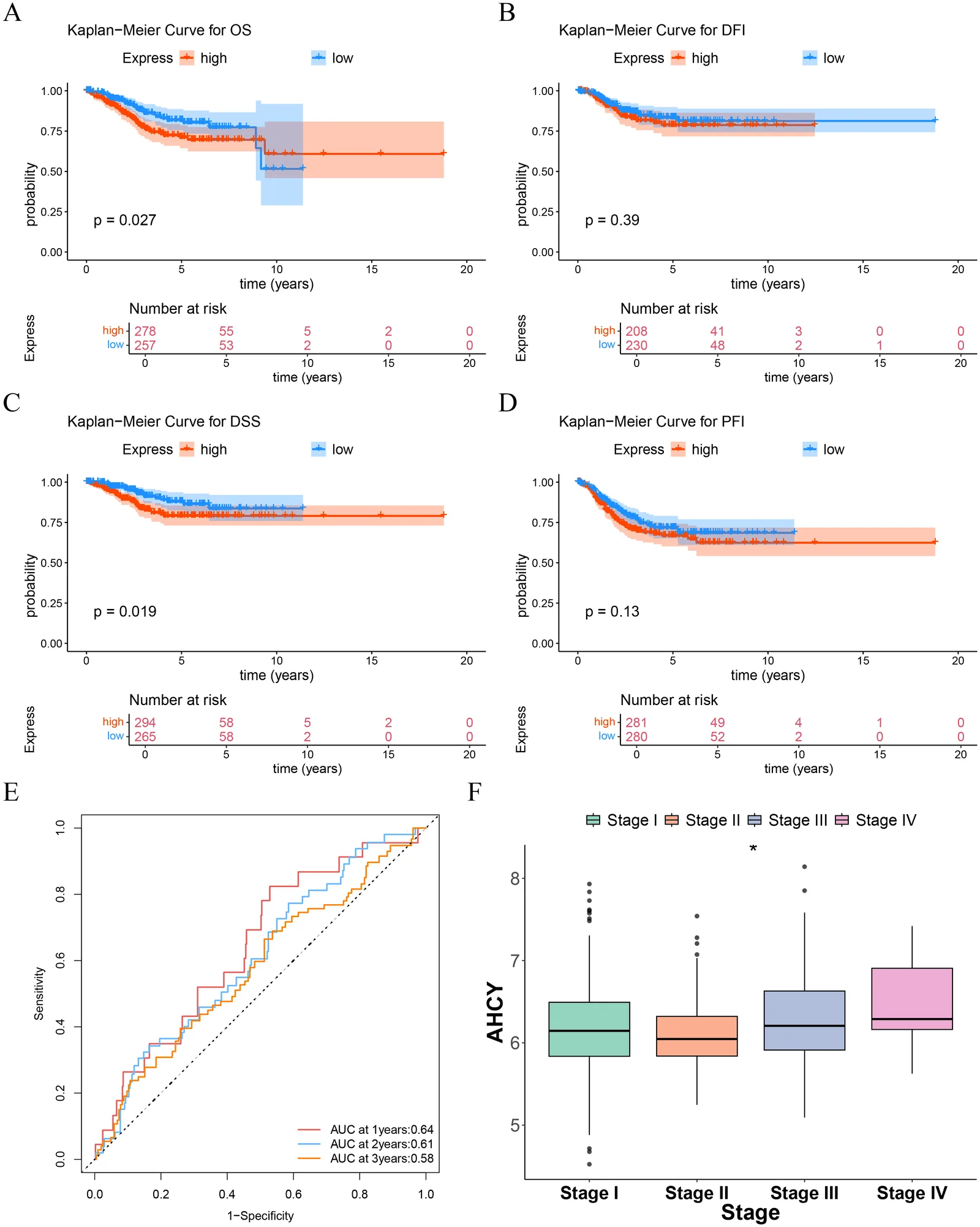
Association of AHCY with cancer prognosis and clinical stage in EC. **(A–D)** Kaplan-Meier analysis of OS **(A)**, and DFI **(B)**, DSS **(C)**, PFI **(D)** in AHCY high- and low-expression groups. **(E)** The operating characteristic (ROC) curves plotted to assess the predictive performance of AHCY expression for 1-, 2-, and 3-year OS in EC patients. **(F)** AHCY expression levels across different tumor stages. *P < 0.05.

In the TCGA-UCEC cohort, patients stratified into the low-AHCY expression cohort demonstrated notably better OS and DSS based on Kaplan-Meier analysis (**Figures 4A–D**). However, no significant differences were observed between the two groups in DFI and PFI. The area under the curve (AUC) values for AHCY in predicting 1-, 2-, and 3-year OS were 0.64, 0.61, and 0.58, respectively (**Figure 4E**). Further assessment of the connection between AHCY expression and EC stage showed statistically noteworthy differences in AHCY expression levels across different tumor stages (**Figure 4F**).

### 3.4 AHCY was implicated in a diverse array of biological processes and signaling pathways

**Figure 5:**
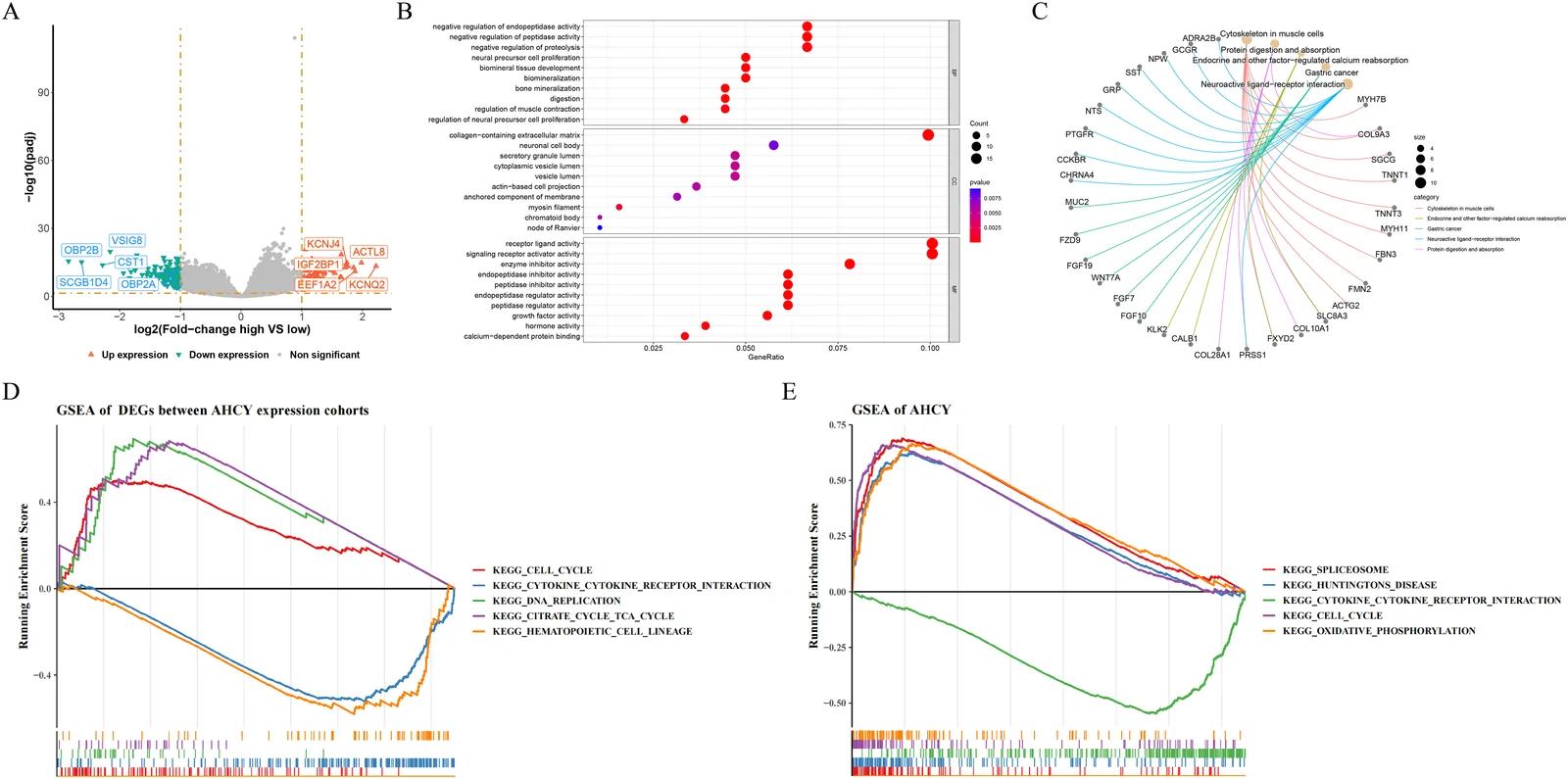
Biological processes and signaling pathways of AHCY. **(A)** Volcano plot of differentially expressed genes (DEGs) between AHCY high- and low-expression groups. **(B)** Top 10 significant Gene Ontology (GO) terms enriched by DEGs. **(C)** Top 5 significant Kyoto Encyclopedia of Genes and Genomes (KEGG) pathways enriched by DEGs. **(D, E)** Top 5 significant pathways enriched by AHCY in gene set enrichment analysis (GSEA).

In total, 269 DEGs between AHCY high- and low-expression groups were identified, comprising 103 up-regulated and 166 down-regulated genes (**Figure 5A**). These DEGs were collectively enriched in 455 GO terms and 24 KEGG pathways (e.g., cytoskeleton in muscle cells) (**Figures 5B, C**; **Supplementary Table 2**). Specifically, the GO analysis revealed 360 significant terms in biological process (BP, e.g., bone mineralization), 37 in cellular component (CC, e.g., myosin filament), and 58 in molecular function (MF, e.g., receptor ligand activity). Subsequent GSEA of the DEGs revealed that AHCY-associated genes were significantly enriched in 20 signaling pathways, including cell cycle (up) and DNA replication (up) among others (**Figure 5D**; **Supplementary Table 3**). Moreover, 113 enriched pathways were related to AHCY expression, with notable downregulation in cytokine-cytokine receptor interaction and upregulation in oxidative phosphorylation (**Figure 5E**; **Supplementary Table 3**).

### 3.5 AHCY was related to the immune microenvironment

**Figure 6:**
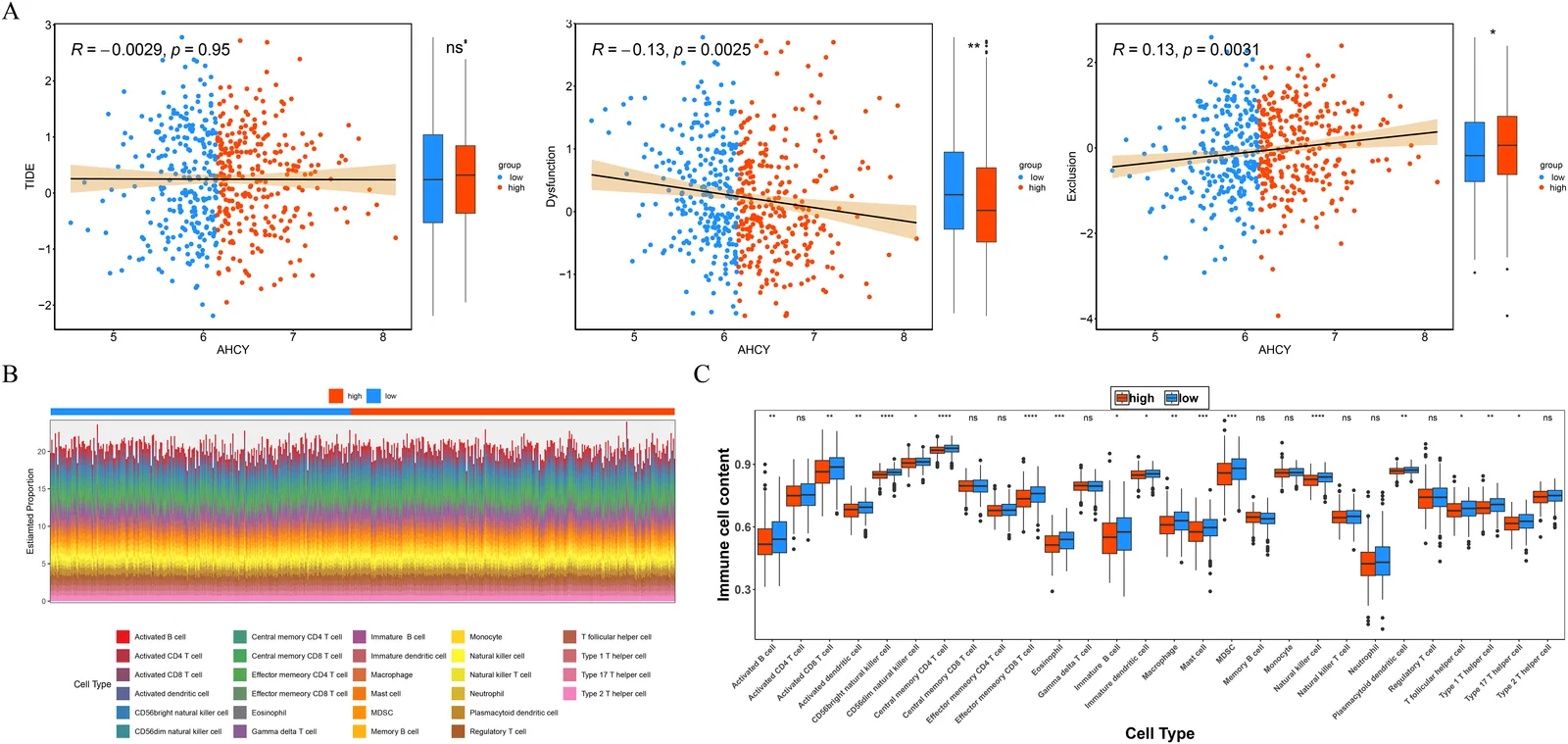
Association of AHCY with immune microenvironment in EC. **(A)** TIDE analysis between the high- and low-AHCY expression cohorts. **(B)** Immune cell infiltration in high- and low-AHCY expression samples. **(C)** Box plot showing the difference in immune cell infiltration between the high- and low-AHCY expression cohorts. ns, not significant; *P < 0.05, **P < 0.01, ***P < 0.001, ****P < 0.0001.

TIDE analysis revealed significantly different scores for both T-cell dysfunction and exclusion between the two AHCY expression cohorts (**Figure 6A**). Specifically, high AHCY expression was associated with increased T-cell exclusion, whereas low expression correlated with a T-cell dysfunction phenotype. Furthermore, 18 immune cell types exhibited notable differences in infiltration between the two expression cohorts, with all showing elevated infiltration in the low-expression group (**Figures 6B, C**).

### 3.6 Single-cell analysis identified macrophages as a potentially relevant AHCY-expressing cell population in EC

**Figure 7:**
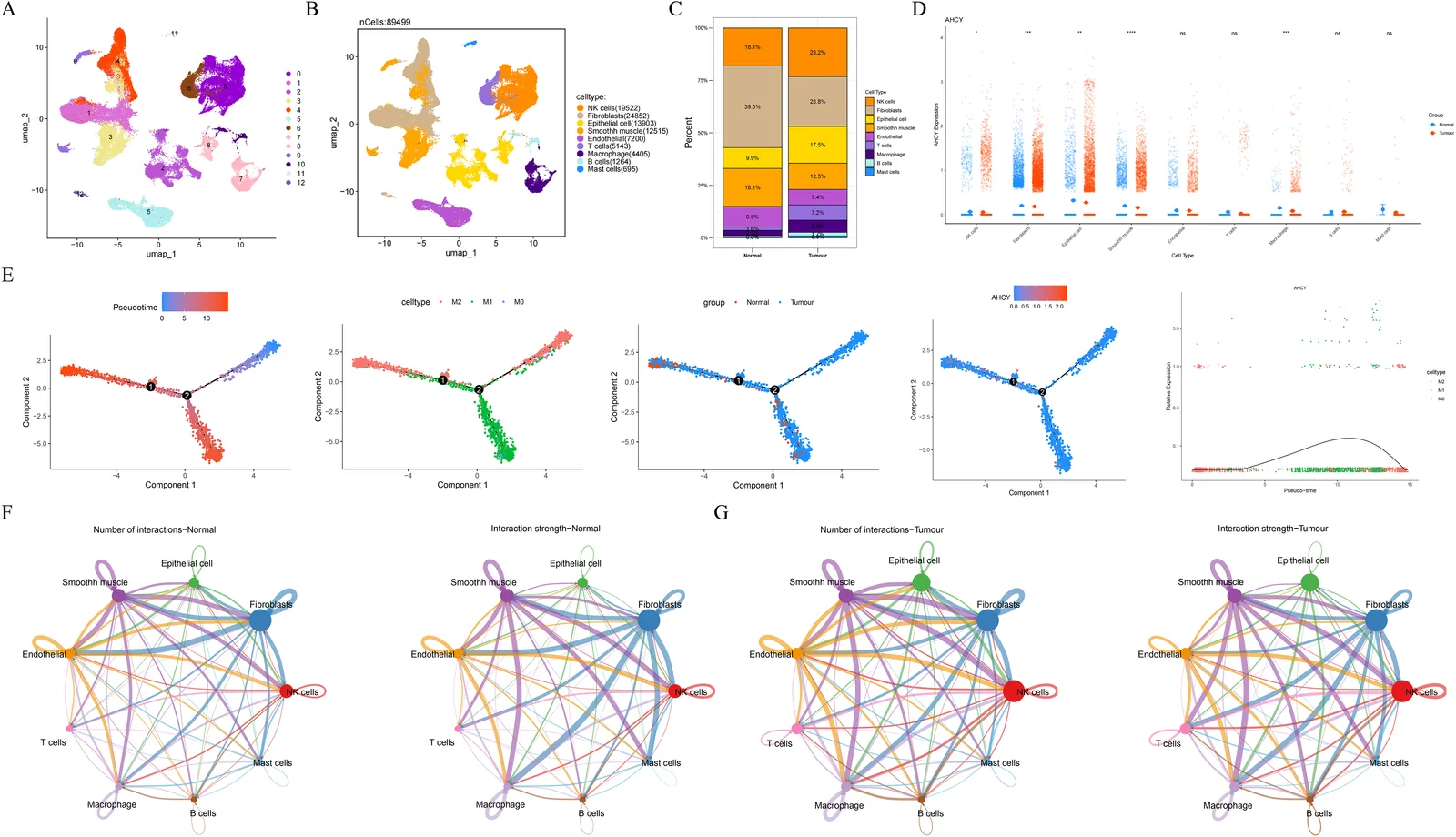
Macrophages dynamics and cell-cell communication in EC. **(A)** Uniform manifold approximation and projection (UMAP) clustering plot of 13 clusters in the single-cell RNA sequencing (scRNA-seq) dataset. **(B)** UMAP plot illustrating the distribution of 9 cell types. **(C)** The cellular composition of each cell type in EC and control groups. **(D)** The expression of AHCY in the EC and control groups. **(E)** From left to right: Time trajectory of macrophages in pseudotime trajectory analysis; Cell subset distribution of macrophages in pseudotime trajectory analysis; Time trajectory of macrophages in the EC group and the normal group; AHCY expression in pseudotime trajectory analysis. **(F)** Cellular communication network illustrating the number (left) and strength (right) of interactions among cell types in the normal group. **(G)** Cellular communication network illustrating the number (left) and strength (right) of interactions among cell types in the EC group. ns, not significant; *P < 0.05, **P < 0.01, ***P < 0.001, ****P < 0.0001.

Following quality control, the top 30 PCs were selected from the PCA based on the top 2,000 HVGs for cell clustering, resulting in the identification of 13 distinct clusters (**Figure 7A**; **Supplementary Figures 1A–D**). Nine distinct cell types were identified based on established marker genes: natural killer (NK) cells, fibroblasts, epithelial cells, smooth muscle cells, endothelial cells, T cells, macrophages, B cells, and mast cells (**Figure 7B**; **Supplementary Figure 1E**). Fibroblasts constituted the largest proportion of cells in the samples, followed by NK cells and epithelial cells (**Figure 7C**). The removal of doublets led to markedly improved resolution, with clearer boundaries between distinct cell populations (**Supplementary Figure 1F**). The selection of macrophages as the focus of this study (key cell type) was driven by their established importance in cancer progression and the marked difference in AHCY expression levels observed between EC and control samples within this cell type. (**Figure 7D**). During the pseudotime trajectory analysis, macrophages differentiated into M0, M1, and M2 subtypes. M2 macrophages were predominantly concentrated at both the beginning and the end of the differentiation trajectory, whereas M1 macrophages accumulated predominantly in the terminal phase. M0 macrophages were sparsely distributed at both the initiation and termination points. Tumor-derived cells were present throughout the entire differentiation spectrum. AHCY expression displayed a biphasic pattern, initially increasing and then decreasing during this process, with predominant expression observed in the M1 macrophage subset (**Figure 7E**). Cell-cell communication analysis revealed enhanced interactions between macrophages and both smooth muscle cells and endothelial cells in tumor samples (**Figures 7F, G**). These interactions were predominantly mediated by the SPP1−CD44 and NAMPT−INSR ligand-receptor pairs, respectively (**Supplementary Figures 1G, H**).

### 3.7 High AHCY expression facilitated EC progression

**Figure 8:**
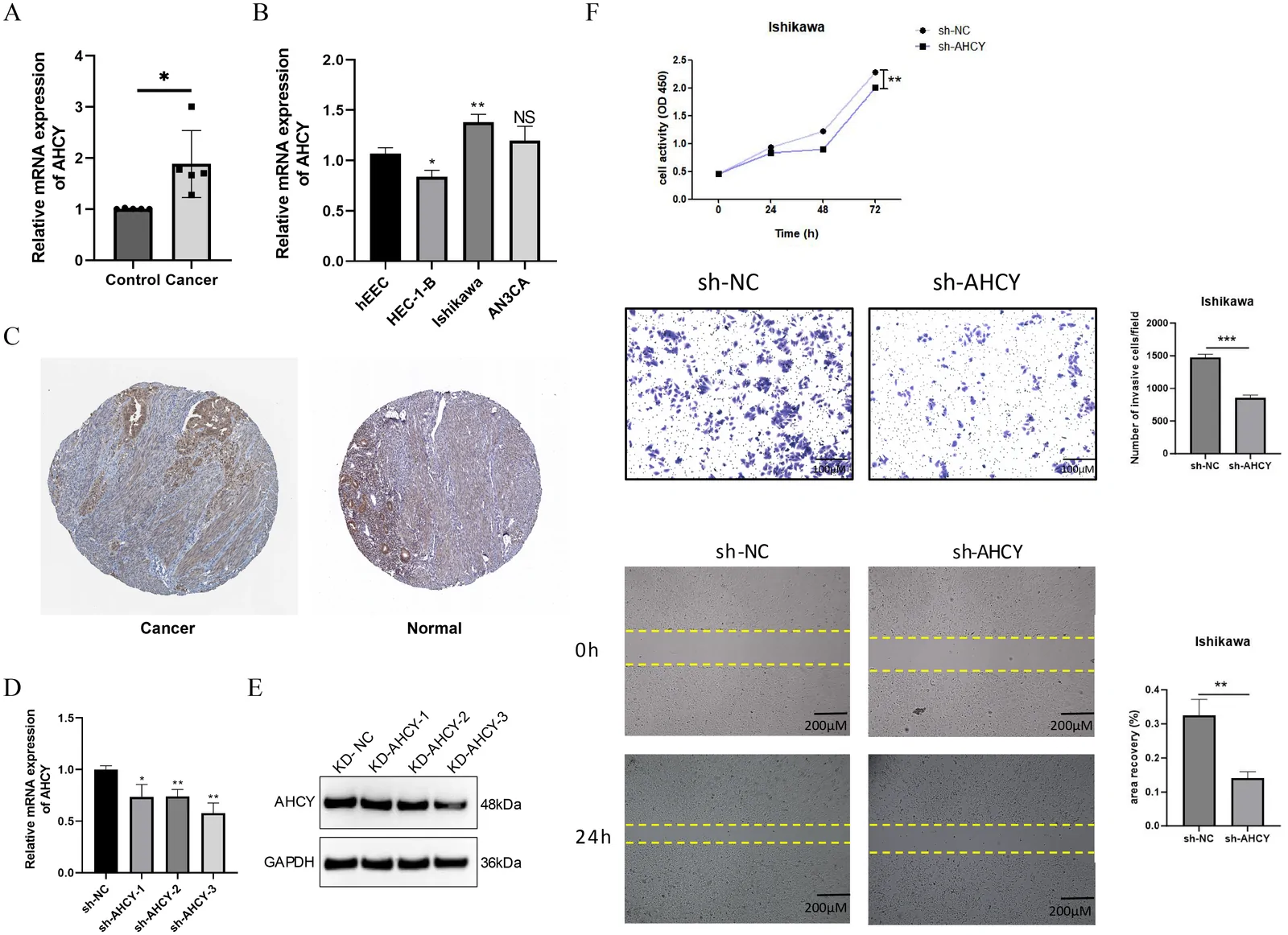
High expression of AHCY facilitates the progression of EC. **(A)** The mRNA expression level of AHCY in clinical EC tissues compared with paired adjacent normal tissues. **(B)** The mRNA expression level of AHCY in human endometrial epithelial cells (hEEC) and the human EC cell lines (Ishikawa, HEC-1-B, and AN3CA). **(C)** Immunohistochemical (IHC) validation of AHCY expression (HPA database). **(D)** The mRNA expression level of AHCY in knockdown Ishikawa cells. **(E)** The protein expression level of AHCY in knockdown Ishikawa cells. **(F)** From top to bottom: Cell proliferation, invasion and migration of Ishikawa cells with AHCY knockdown. NS, not significant; *P < 0.05, **P < 0.01, ***P < 0.001, ****P < 0.0001.

The mRNA expression level of AHCY was markedly upregulated in clinical EC tissues compared with paired adjacent normal tissues (**Figure 8A**). Differential AHCY expressions were observed in EC cell lines compared to normal human cells (hEEC): it was down-regulated in HEC-1-B cells, up-regulated in Ishikawa cells, and showed no statistically significant difference in AN3 CA cells (**Figure 8B**). IHC images obtained from the Human Protein Atlas (HPA) database showed weak AHCY staining in normal endometrial tissues and moderate staining in EC tissues (**Figure 8C**). The AHCY expression pattern in Ishikawa cells was consistent with that observed in EC tissues, justifying its selection as a representative model for subsequent functional experiments. Consistently, DepMap CRISPR screening showed negative AHCY Chronos scores across all 34 uterine-derived cell lines, including 28 endometrial carcinoma cell lines, with a median score of -0.936 and a range of -1.449 to -0.267. The Ishikawa-derived cell line had a score of -0.970, supporting AHCY dependency across multiple endometrial cancer cell-line backgrounds (**Supplementary Table 4**). Three distinct shRNAs (sh-AHCY-1, sh-AHCY-2, and sh-AHCY-3) were utilized to knock down AHCY in Ishikawa cells. All knock-down groups showed a substantial reduction in AHCY mRNA and protein levels when compared to the control group, with sh-AHCY-3 demonstrating the highest silencing efficiency (**Figures 8D, E**). Given that sh-AHCY-3 demonstrated the most potent knockdown effect, it was utilized in all further investigations. Compared with the control group, AHCY knockdown in Ishikawa cells resulted in markedly attenuated cell viability, migration, and invasion capabilities (**Figure 8F**). In conclusion, the above results indicated AHCY overexpression contributes to EC progression by enhancing malignant behaviors.

### 3.8 AHCY knockdown promoted apoptosis and induced cell cycle dysregulation in Ishikawa cells

**Figure 9:**
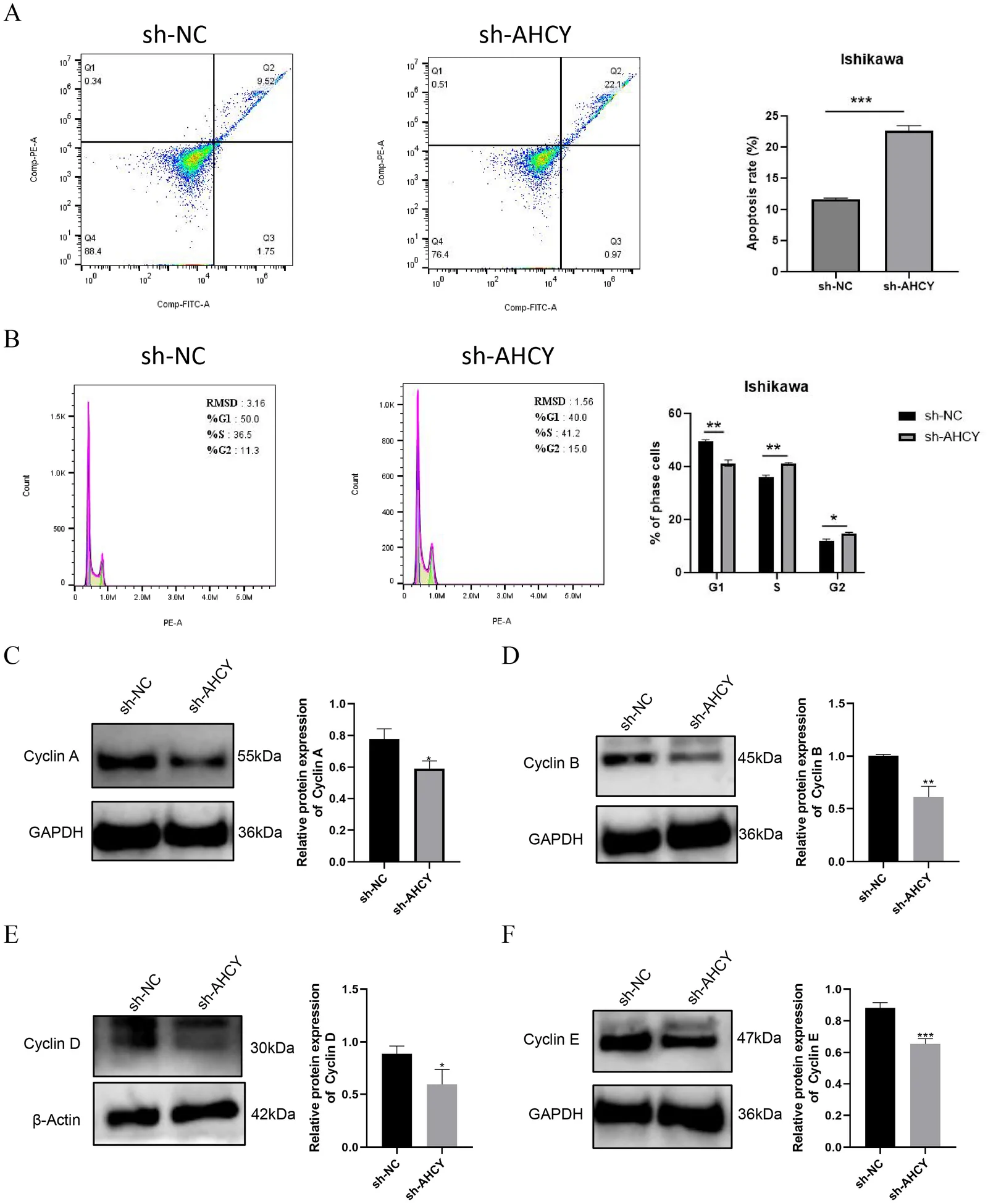
Apoptosis and cell cycle in AHCY knockdown Ishikawa cells. **(A)** Cell apoptosis in AHCY knockdown Ishikawa cells. **(B)** Cell cycle in AHCY knockdown Ishikawa cells. **(C–F)** The expression levels of cyclins A **(C)**, B **(D)**, D **(E)**, and E **(F)** in AHCY knockdown Ishikawa cells. *P < 0.05, **P < 0.01, ***P < 0.001.

Knockdown of AHCY observably increased the apoptosis rate in Ishikawa cells. This indicates that high expression of AHCY normally acts to suppress apoptotic cell death in endometrial cancer cells (**Figure 9A**). AHCY depletion led to a significant arrest of the cell cycle, characterized by a decrease in the proportion of cells in the G1 phase and a concomitant increase in the S and G2/M phases (**Figure 9B**). A notable downregulation in the expression levels of important cyclins, such as cyclins A, B, D, and E, further supported this arrest (**Figures 9C–F**). In summary, AHCY knockdown promotes apoptosis and induces cell cycle arrest in Ishikawa cells, providing direct functional evidence that high AHCY expression contributes to EC progression by inhibiting cell death and facilitating uncontrolled cell cycle disorder.

### 3.9 Folic acid suppressed Ishikawa cell proliferation and reduced AHCY expression

**Figure 10:**
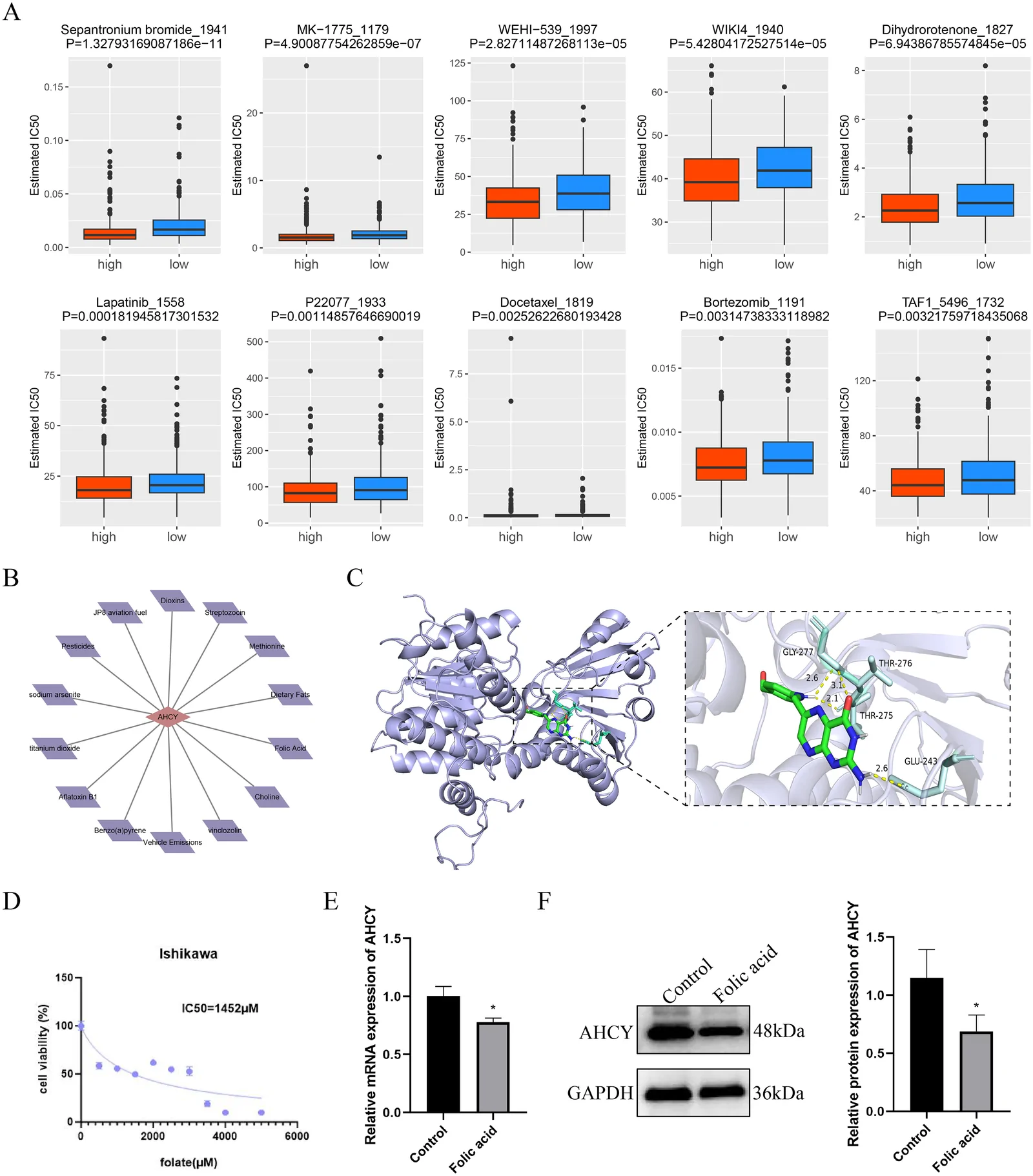
Drug sensitivity analysis and drug prediction. **(A)** Half-maximal inhibitory concentration (IC_50_) values of top 10 significant drugs between the high- and low-AHCY expression cohorts. **(B)** Gene-drug interaction network. **(C)** Molecular docking of AHCY with folic acid. **(D)** The effect of folic acid on Ishikawa cell proliferation. **(E, F)** The expression of AHCY in Ishikawa cells treated by folic acid. *P < 0.05.

Drug sensitivity analysis identified 24 drugs, including Sepantronium bromide (1941), MK-1775 (1179), WEHI-539 (1997), and WIKI4 (1940), whose efficacy was significantly associated with AHCY expression levels, highlighting them as potential therapeutic options for stratified treatment (**Figure 10A**; **Supplementary Table 5**). Thereafter, 14 compounds were predicted to target AHCY computationally (**Figure 10B**). Among these, folic acid as a non-toxic candidate was selected for further validation via molecular docking. The binding mode of folic acid to AHCY involved critical hydrogen bonds with residues such as GLY-277 and GLU-243 (**Figure 10C**). This interaction was characterized by a high binding affinity, as evidenced by a free energy value of -9.6 kcal/mol. Moreover, folic acid exerted a marked anti-proliferative effect on Ishikawa cells, demonstrating dose-dependent growth inhibition and an IC50 value of 1,452 µM, which suggests its potential as a therapeutic agent (**Figure 10D**). Folic acid treatment reduced AHCY expression at both the mRNA and protein levels in Ishikawa cells (**Figures 10E, F**). However, whether this effect involves direct targeting of AHCY remains to be determined.

## 4 Discussion

Our study examined the clinical and biological significance of AHCY in EC. Integrating pan-cancer profiling, bulk/scRNA-seq, and bench validation, we found AHCY aberrantly expressed in EC tissues and cell lines, with higher levels linked to advanced stage and worse survival. Functionally, AHCY promoted EC progression by activating cell-cycle/DNA-replication programs and restraining apoptosis: knockdown reduced proliferation, migration/invasion, and induced cell-cycle arrest. Immune analyses associated AHCY with reduced infiltration and T-cell exclusion, and highlighted macrophages as a key AHCY-expressing population. Drug-sensitivity modeling and docking prioritized folic acid as a putative AHCY inhibitor; we confirmed anti-proliferative activity with concomitant AHCY downregulation *in vitro*. Collectively, these data nominate AHCY as a candidate prognostic biomarker and therapeutic target in EC. Evidence from previous studies suggests that AHCY is involved in the progression of multiple cancer types. In glioblastoma (GBM), MAT2A and AHCY are identified as essential enzymes by pan-cancer CRISPR profiling, their blockade impairing oxidative metabolism and shifting cells toward oxidative stress and death (27). In AOM/DSS-induced colitis-associated colon cancer, AHCY is overexpressed relative to adjacent mucosa, correlating with Hedgehog pathway activation and aggressive phenotypes (28). In colorectal cancer, AHCY is upregulated versus matched normal mucosa, associated with APC loss, disruption of the methionine cycle, and heightened crypt proliferation (29). In esophageal squamous cell carcinoma (ESCC), AHCY activated by heat-induced STIP1 binds LDHA and recruits PRMT3 to methylate LDHA (R106), thereby enhancing glycolysis, preventing ubiquitination-mediated degradation of AHCY, and ultimately promoting tumor growth and sustaining tumor metabolism (30). In bladder urothelial carcinoma (BLCA), inhibiting AHCY effectively suppresses the proliferation, migration, and invasion of BLCA (31). In this study, we found and verified the high expression of AHCY in EC, and knockdown of AHCY inhibited the proliferation, migration and invasion of EC cells, while promoting apoptosis. These findings collectively underscore AHCY as a critical regulator that sustains tumor growth and aggressiveness across diverse cancer types, positioning it as a promising therapeutic target for precision oncology.

To systematically elucidate the multidimensional biological functions of AHCY in EC, we conducted a comprehensive functional analysis. Results demonstrated that in EC, AHCY displayed significant positive correlation with four DNA methyltransferases and co-expression with RNA-modification regulators. In patient specimens, m6A profiling demonstrated a significant increase in m6A modification in EC compared with paired noncancerous tissues. AHCY clears SAH to preserve the SAM: SAH ratio, thereby providing the metabolic basis for DNA and RNA methylation (8, 32). Moreover, the occurrence and development of EC involve complex epigenetic networks encompassing DNA methylation, RNA modifications, histone modifications, and chromatin remodeling (33, 34). These findings reveal the pivotal role of AHCY in the epigenetic regulation of EC, highlighting its potential as a key therapeutic target for intervening in EC-associated epigenetic dysregulation.

Functionally, AHCY might promote EC progression by activating cell-cycle/DNA-replication programs. Aberrations in cell-cycle progression constitute a fundamental mechanism of tumorigenesis (35). AHCY knockdown and adenosine depletion induce DNA damage and cell-cycle arrest (13). We computed cell-cycle activity by ssGSEA and observed significant tumor–normal differences across cancer types, particularly in endometrial cancer (EC). In EC, AHCY expression correlated moderately with cell-cycle activity (r = 0.58, *P* < 0.05), with similar associations across multiple tumors, including but not limited to GBM and ovarian cancer OV. We assessed cell-cycle distribution in EC cells by flow cytometry. AHCY knockdown decreased the G1 fraction while increasing S and G2/M fractions. Consistently, key cyclins (Cyclin A, B, D, and E) were markedly downregulated, supporting this arrest-like phenotype. Collectively, AHCY depletion promoted apoptosis and induced cell-cycle delay/accumulation in Ishikawa cells, providing functional evidence that elevated AHCY facilitates endometrial cancer progression by restraining cell death and fostering cell-cycle dysregulation.

To explore the biological functions of AHCY, we identified AHCY co-expressed genes and performed GO, KEGG, and GSEA enrichment analyses. Despite methodological differences among these approaches, convergent signals implicated AHCY in DNA replication, cell-cycle regulation, oxidative phosphorylation, and multiple cancer-related pathways in EC.

Immunotherapy has introduced both complexity and promise to the therapeutic landscape of EC (36). TIDE analysis revealed significant differences in T-cell dysfunction and exclusion between AHCY expression cohorts. Specifically, higher AHCY levels were associated with increased T-cell exclusion, whereas lower AHCY levels corresponded to a T-cell dysfunction phenotype. Moreover, infiltration of 18 immune cell types differed significantly between groups, with uniformly higher infiltration in the AHCY-low cohort. These findings suggest that AHCY-high tumors may exhibit an immune-excluded microenvironment.

Tumor-associated macrophages (TAMs) are a key component of the tumor microenvironment and have emerged as therapeutic targets in cancer (37). In EC, the tumor microenvironment regulates the recruitment and polarization of TAMs, resulting in an infiltration predominantly composed of M2-like macrophages (38). In scRNA-seq analysis, macrophages were prioritized; pseudotime resolved M0–M1/M2 differentiation, AHCY showed biphasic expression peaking in M1, and cell–cell communication analysis revealed strengthened macrophage–stromal/endothelial crosstalk via SPP1–CD44 and NAMPT–INSR axes. These findings suggest that AHCY expression is associated with macrophage states and macrophage–stromal interactions in EC. However, macrophage-specific functional studies are required to establish causality.

Finally, molecular docking identified folic acid as a putative AHCY inhibitor. folic acid suppressed Ishikawa cell proliferation in a dose-dependent manner and concomitantly downregulated AHCY mRNA and protein, consistent with AHCY-targeted growth inhibition. The metabolism of folic acid and its dependent enzymes play a crucial role in DNA synthesis and methylation processes (39). Taken together, these findings identify folic acid as a potential AHCY-associated candidate with anti-proliferative effects in EC cells. However, the precise mechanism by which folic acid-mediated AHCY inhibition suppresses EC growth—whether through direct allosteric regulation of AHCY, depletion of methyl-donor pools, or interference with downstream methylation-dependent transcriptional programs—requires further mechanistic investigation.

However, several limitations of this study should be acknowledged. First, a substantial proportion of our findings was derived from retrospective public datasets and may therefore be affected by selection bias, cohort heterogeneity, and incomplete clinical information. Second, many of the observed associations—such as those between AHCY expression, epigenetic markers, and immune—are correlative and do not establish direct causality. Third, the analysis relied on a relatively limited number of clinical specimens, which may constrain the generalizability of the findings across diverse EC subtypes or patient populations. Fourth, although DepMap CRISPR screening supported AHCY dependency across multiple endometrial cancer cell lines, our functional experiments were restricted to Ishikawa cells, and direct experimental validation in additional cell lines and *in vivo* models is still required. Moreover, although AHCY knockdown inhibited malignant phenotypes and altered cell-cycle progression, the underlying molecular mechanisms remain unclear, and the present findings primarily describe phenotypic rather than mechanistic effects. Fifth, inferences regarding cell-type-specific functions drawn from single-cell transcriptomic data lack spatial validation through techniques such as multiplex immunohistochemistry or spatial transcriptomics, leaving the tissue-contextual interactions unresolved. Finally, while molecular docking and *in vitro* assays suggest folic acid as a putative AHCY inhibitor with anti-proliferative effects, this hypothesis remains preliminary without confirmation through *in vivo* pharmacokinetic, efficacy, or toxicity studies. Future work should aim to address these gaps through mechanistic experiments, expanded cohorts, multimodal spatial analyses, and pre-clinical *in vivo* validation.

## 5 Conclusions

This study identified AHCY as a potential oncogenic factor in EC, with its expression associated with cell-cycle, epigenetic, and immune microenvironment alterations. Concurrently, we observed that the anti-proliferative activity of folic acid was accompanied by reduced AHCY expression, although their direct mechanistic relationship requires further validation. Future efforts should focus on establishing AHCY’s value as a precision therapy target for EC through in-depth mechanistic studies, multi-model validation, and clinical translation exploration.

## Data Availability

The original contributions presented in the study are included in the article/**Supplementary Material**. Further inquiries can be directed to the corresponding author/s

## Glossary

EC: Endometrial cancer
AHCY: S-adenosylhomocysteine hydrolase
SAH: S-adenosylhomocysteine
SAM: S-adenosylmethionine
UCSC: University of California Santa Cruz
OS: Overall survival
ScRNA-seq: Single-cell RNA sequencing
CCLE: Cancer Cell Line Encyclopedia
DSS: Disease-specific survival
DFI: Disease-free interval
CNA: Copy number alteration
MSI: Microsatellite instability
TMB: Tumor mutational burden
ROC: Receiver operating characteristic
DEGs: Differentially expressed genes
KEGG: Kyoto encyclopedia of genes and genomes
GO: Gene ontology
GSEA: Gene set enrichment analysis
TIDE: Tumor immune dysfunction and exclusion
HPA: Human protein atlas
HVGs: Highly variable genes
PCA: Principal Component Analysis
PCs: Principal components
UMAP: Uniform manifold approximation and projection
CTD: Comparative toxicogenomics database
PDB: Protein data bank
hEEC: Human endometrial epithelial cells
RT-qPCR: Reverse transcription-quantitative polymerase chain reaction
shRNA: Short hairpin RNA
COAD: Colon adenocarcinoma
RCC: Renal cell carcinoma
PAAD: Pancreatic adenocarcinoma
AUC: Area under the curve
BP: Biological process
CC: Cellular component
MF: Molecular function
GBM: Glioblastoma
ESCC: Esophageal squamous cell carcinoma
BLCA: Bladder urothelial carcinoma
TAMs: Tumor-associated macrophages
