# Supplementary material for: Integrated bulk and single-cell transcriptomics unveil the role of AHCY in cell cycle regulation of endometrial cancer
Source: Front Oncol. 2026 Aug 20;16:1770032. doi: 10.3389/fonc.2026.1770032 (PMC13538023; doi:10.3389/fonc.2026.1770032)
Supplement: Supplementary file 1 [file Table1.docx]

Figure S1. scRNA-seq data processing (A) scRNA-seq data before (left) and after (right) quality control. (B) Selection of top 2,000 highly variable genes (HVGs). (C) Elbow plot of principal components (PCs). (D) Permutation test of PCs. (E) Expression of marker genes in the main cell types. (F) The proportion of doublets among various cell types. (G) Ligand–receptor pairs of cell types in the normal group. (H) Ligand–receptor pairs of cell types in the EC group.

Table S1 Lists of primer sequence

| Primer | Sequence |
| --- | --- |
| GAPDH | F´: 5-CTGGGCTACACTGAGCACC-3´ |
|  | R: 5´-AAGTGGTCGTTGAGGGCAATG-3´ |
| AHCY | F:5´-ATTCCGGTGTATGCCTGGAAG-3´ |
|  | R:5´-GAGATGCCTCGGATGCCTG-3´ |
| sh-AHCY-1 | F:5´-CCGGATGCCATTGTGTGTAACATTGCTCGAGCAATGTTACACACAATGGCATTTTTTG-3´ |
|  | R:5´-AATTCAAAAAATGCCATTGTGTGTAACATTGCTCGAGCAATGTTACACACAATGGCAT-3´ |
| sh-AHCY-2 | F:5´-CCGGGTCAGGAGGGCAACATCTTTGCTCGAGCAAAGATGTTGCCCTCCTGACTTTTTG-3´ |
|  | R:5´-AATTCAAAAAGTCAGGAGGGCAACATCTTTGCTCGAGCAAAGATGTTGCCCTCCTGAC-3´ |
| sh-AHCY-3 | F:5´-CCGGCCGTCACCAAGAGCAAGTTTGCTCGAGCAAACTTGCTCTTGGTGACGGTTTTTG-3´ |
|  | R:5´-AATTCAAAAACCGTCACCAAGAGCAAGTTTGCTCGAGCAAACTTGCTCTTGGTGACGG-3´ |
